# An Overview of Plant Phenolic Compounds and Their Importance in Human Nutrition and Management of Type 2 Diabetes

**DOI:** 10.3390/molecules21101374

**Published:** 2016-10-15

**Authors:** Derong Lin, Mengshi Xiao, Jingjing Zhao, Zhuohao Li, Baoshan Xing, Xindan Li, Maozhu Kong, Liangyu Li, Qing Zhang, Yaowen Liu, Hong Chen, Wen Qin, Hejun Wu, Saiyan Chen

**Affiliations:** 1 College of Food Science, Sichuan Agricultural University, No. 46, Xinkang Road, Ya’an 625014, Sichuan, China; 18227591863@126.com (M.X.); 18227591123@126.com (J.Z.); sakataharuka@hotmail.com (Z.L.); 18227589580@163.com (X.L.); 18227589574@163.com (M.K.); 18227584828@163.com (L.L.); zhangqing@sicau.edu.cn (Q.Z.); lyw@my.swjtu.edu.cn (Y.L.); chenhong945@sicau.edu.cn (H.C.); qinwen@sicau.edu.cn (W.Q.); hejunwu520@163.com (H.W.); 13551570482@163.com (S.C.); 2 Stockbridge School of Agriculture, University of Massachusetts, Amherst, MA 01003, USA; bx@umass.edu

**Keywords:** phenolic compounds, biosynthesis, function, complication, type 2 diabetes

## Abstract

In this paper, the biosynthesis process of phenolic compounds in plants is summarized, which include the shikimate, pentose phosphate and phenylpropanoid pathways. Plant phenolic compounds can act as antioxidants, structural polymers (lignin), attractants (flavonoids and carotenoids), UV screens (flavonoids), signal compounds (salicylic acid and flavonoids) and defense response chemicals (tannins and phytoalexins). From a human physiological standpoint, phenolic compounds are vital in defense responses, such as anti-aging, anti-inflammatory, antioxidant and anti-proliferative activities. Therefore, it is beneficial to eat such plant foods that have a high antioxidant compound content, which will cut down the incidence of certain chronic diseases, for instance diabetes, cancers and cardiovascular diseases, through the management of oxidative stress. Furthermore, berries and other fruits with low-amylase and high-glucosidase inhibitory activities could be regarded as candidate food items in the control of the early stages of hyperglycemia associated with type 2 diabetes.

## 1. Introduction

**Figure 1:**
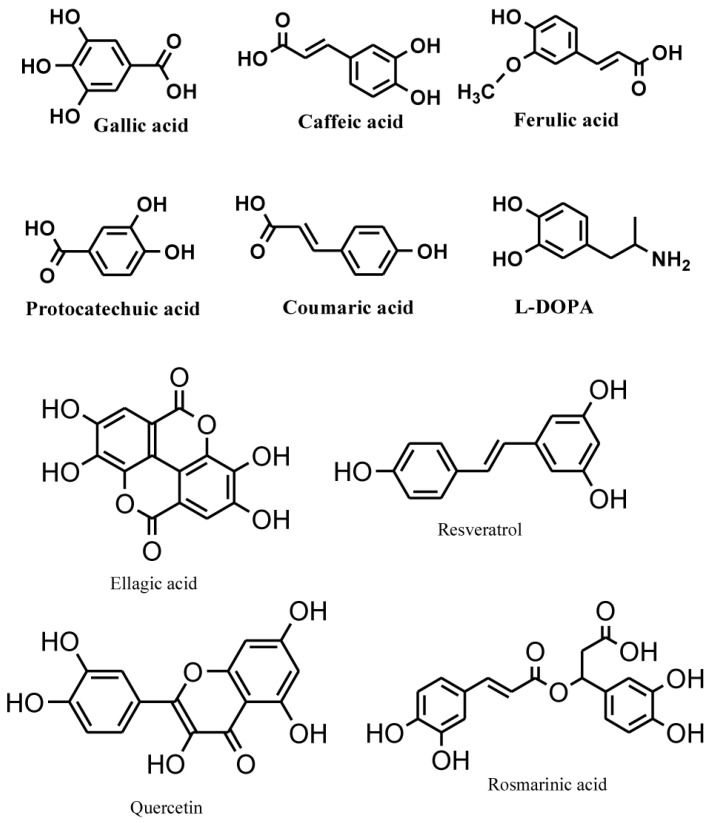
Common phenolic compounds in plants comprise an aromatic ring, bear one or more hydroxyl substituents and range from simple phenolic molecules to highly polymerized compounds (modified from Velderrain-Rodríguez et al., 2014) [2].

**Figure 2:**
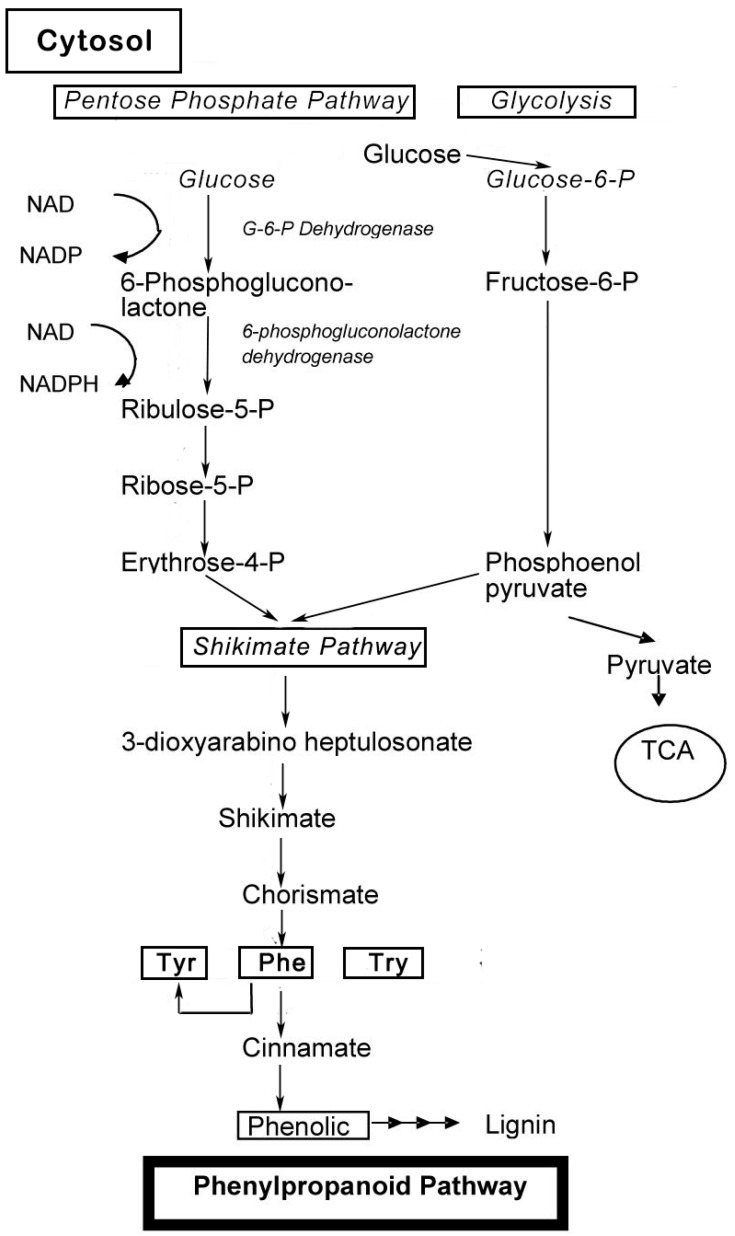
Biosynthesis of phenol compounds in the pentose phosphate, shikimate and phenylpropanoid pathways in plants (modified from Vattem et al., 2005, and Lin et al., 2010) [3,4].

Phenolic compounds are secondary metabolites, which are produced in the shikimic acid of plants and pentose phosphate through phenylpropanoid metabolization [1]. They contain benzene rings, with one or more hydroxyl substituents, and range from simple phenolic molecules to highly polymerized compounds [2] (Figure 1). In the synthesis of phenolic compounds, the first procedure is the commitment of glucose to the pentose phosphate pathway (PPP) and transforming glucose-6-phosphate irreversibly to ribulose-5-phosphate. The first committed procedure in the conversion to ribulose-5-phosphate is put into effect by glucose-6-phosphate dehydrogenase (G6PDH). On the one hand, the conversion to ribulose-5-phosphate produces reducing equivalents of nicotinamide adenine dinucleotide phosphate (NADPH) for cellular anabolic reactions. On the other hand, PPP also produces erythrose-4-phosphate along with phosphoenolpyruvate from glycolysis, which is then used through the phenylpropanoid pathway to generate phenolic compounds after being channeled to the shikimic acid pathway to produce phenylalanine [3,4] (Figure 2). Phenolics are the most pronounced secondary metabolites found in plants, and their distribution is shown throughout the entire metabolic process. These phenolic substances, or polyphenols, contain numerous varieties of compounds: simple flavonoids, phenolic acids, complex flavonoids and colored anthocyanins [5] (Figure 1). These phenolic compounds are usually related to defense responses in the plant. However, phenolic metabolites play an important part in other processes, for instance incorporating attractive substances to accelerate pollination, coloring for camouflage and defense against herbivores, as well as antibacterial and antifungal activities [6,7,8].

Phenolic compounds, including stress-linked phytochemicals, have been related to favorable impacts, which are caused by the consumption of fruits and vegetables, particularly due to their antioxidant activity [9]. Balasundram et al., (2006) reviewed [10] the antioxidant activity, occurrence and latent uses of phenolic compounds in plants and agri-industrial by-products. Under those reports, fruits, vegetables and beverages are the principle sources of phenolic compounds in the human diet. Plant polyphenols as dietary antioxidants in human health and disease might protect against oxidative damage. As natural antioxidants, phenolic compounds are found abundantly in plant food and beverages, which play vital parts in pabulum and healthcare. Some research have indicated that phenolic compounds are the most affluent in ordinary human diets among the dietary antioxidants. Lately, phenolic compounds have obtained significant interest based on active reports of their conjectural part in holding back a variety of human illnesses [11,12,13]. It is well-known that normally consumed fresh and processed fruits, for instance raspberries, cranberries, apples, grapes, pears and jams, are the major sources of phenolic compounds, and strawberries and their derived products, like juices [3,14]. This review focuses on the present understanding of the potential efficacy of polyphenols on carbohydrate metabolism and glucose homeostasis, which has been commendably studied in vitro, some clinical experiments and animal models [15].

## 2. Health Benefits of Phenolic Compounds

The chemical constituents extracted from plants, phenolic compounds, can inhibit the absorption of amylase in the treatment of carbohydrate absorption, such as diabetes [16]. There are many fruits and vegetables that contain phenolic compounds, especially, grapes, berries and tomatoes. Phenolic compounds, for instance phenolic acids and flavonoids, could promote health benefits by reducing the risk of metabolic syndrome and the related complications of type 2 diabetes. However, different groups of phenolic compounds have different biological characteristics, and very little is known about the mechanisms by which they could contribute to the prevention of disease; there still is the need for further studies.

**Figure 3:**
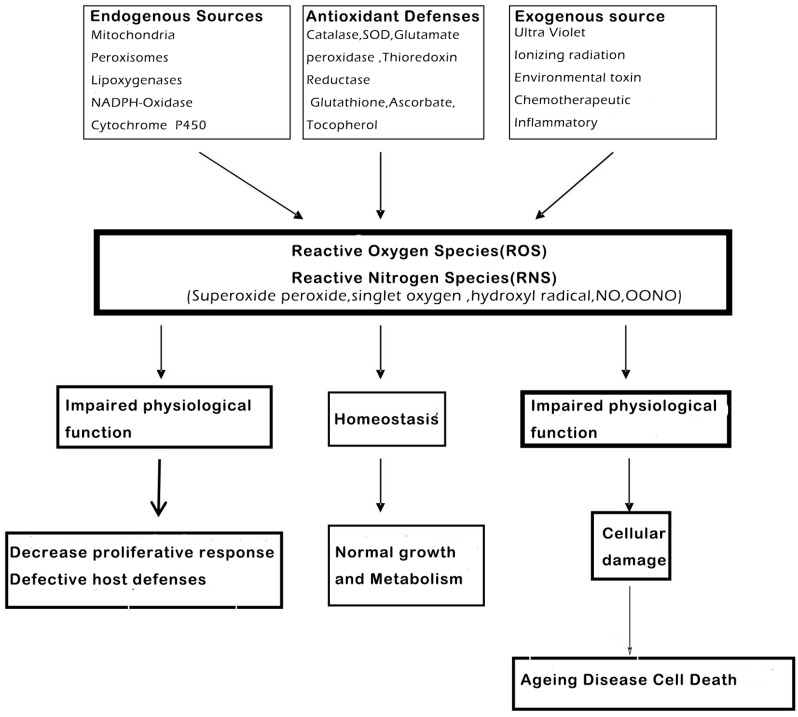
Reactive oxygen (ROS) and nitrogen (RNS) species are highly reactive oxidized molecules, including superoxide, peroxide, singlet oxygen, hydroxyl radical, NO and OONO, that are constantly produced under normal cellular conditions, such as during homeostasis and impaired functions, which could lead to cellular damage, like ageing, disease and cell death (modified from Shetty et al., 2004.) [20].

Reactive oxygen (ROS) and reactive nitrogen species (RNS) are highly reactive oxidized molecules, which are generated constantly by normal cellular conditions, for instance the activity of the mitochondrial respiratory chain and inflammation, which could lead to damage in other biological molecules, like proteins and DNA [17,18,19]. The antioxidant enzymes include superoxide dismutase (SOD), glutathione peroxidase (GPx) and catalase (CAT), all of them will play a vital role in getting rid of these oxidants and preventing cellular injury (Figure 3).

**Figure 4:**
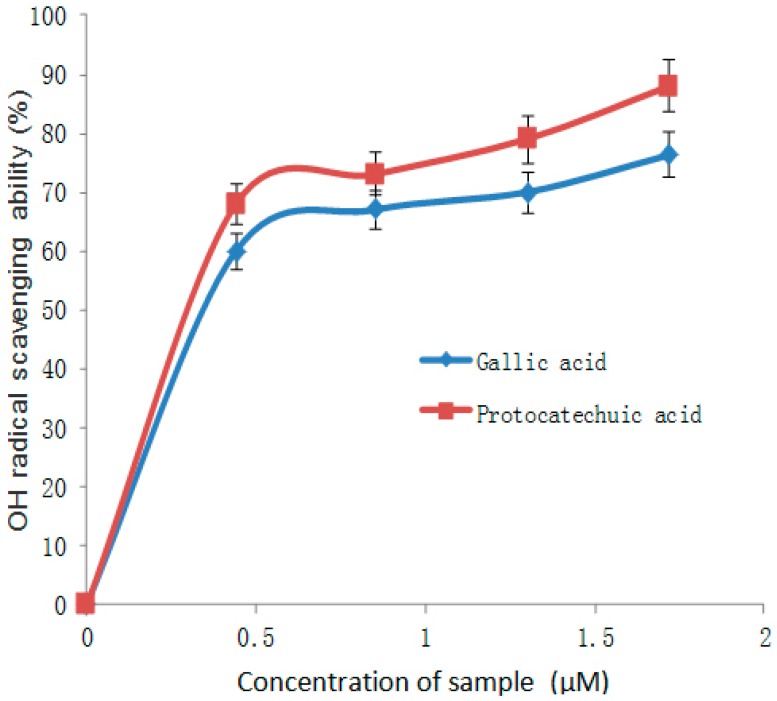
OH radical scavenging ability of gallic and protocatechuic acids.

**Figure 5:**
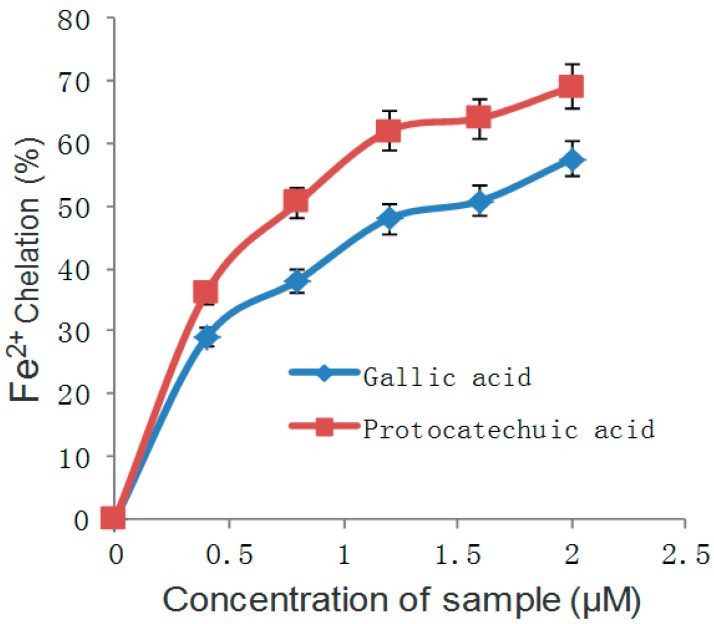
Fe^2+^ chelating ability of gallic and protocatechuic acids.

Many studies have reported the advantages of phenolic compounds, such as anti-aging, anti-inflammatory, antioxidant and antiproliferative agents. In addition to the adjustment of the above, there are relevant antioxidant enzymes to counter oxidants [21,22]. Polyphenols, especially flavonoids, phenolic acids and tannins, have the important property of inhibiting α-glucosidase and α-amylase, which are key enzymes and responsible for the digestion of dietary carbohydrates to glucose. Dietary plant polyphenols and polyphenol-rich products modulate carbohydrate and lipid metabolism, attenuate hyperglycemia, dyslipidemia and insulin resistance, improve β-cell function, stimulate insulin secretion, improve adipose tissue metabolism and alleviate oxidative stress, stress-sensitive signaling pathways and inflammatory processes. Polyphenolic compounds can also prevent the development of long-term diabetes complications, including cardiovascular disease, neuropathy, nephropathy and retinopathy. There is evidence that the small crimson fruit of *Viburnum dilatatum* Thunb has strong antioxidant activity, and cyanidin 3-sambubioside (C3S) and 5-caffeoyl quinic acid (5-CQA) are identified as active compounds, which were orally administered to streptozotocin-induced diabetic rats for four weeks repeatedly. Results are presented as shown in Table 1 [23,24]. From this study, both phenolic acids (gallic and and protocatechuic acids) showed concentration-dependent inhibition of α-amylase and α-glucosidase activities in vitro (Figure 4 and Figure 5). Furthermore, phenolic compounds that are found in beverages, vegetables, galenical pears and berries, may facilitate fitness by decreasing the risk of metabolic syndrome and relevant complications of type 2 diabetes [25].

These effects are attributed in general to the potential ability of the phenolic compounds to reduce, counteract or also repair damage resulting from oxidative stress and inflammation associated with these diseases conditions. Experimental findings from the IC_50_ values for rat intestinal maltase and porcine pancreatic α-amylase showed 334 and 739 μM, respectively, for (−)-3-*O*-galloylepicatechin and 150 and 401 μM, respectively, for (−)-3-*O*-galloylcatechin [26] (Table 2). Molecular biological studies have found that EGCG exhibits a strong inhibitory activity on the proteasome (in vitro activity IC_50_ = 86 nM; in vivo activity IC_50_ = 18 μM) [27]. 

Many studies have associated the increase in the consumption of fruits and vegetables containing high levels of antioxidant compounds with the reduction on the risk of certain chronic diseases, for instance diabetes and cardiovascular diseases [33,34,35]. Some polyphenol-rich foods, for instance grape, grape seed extract, pomegranate juice and cranberry juice, have been reported to play a beneficial part in reducing cardiovascular risk elements in patients with metabolic syndrome and type 2 diabetes. There is a recent study that aimed to test the hypothesis that grapeseed extract (GSE) may improve these markers in high-risk cardiovascular subjects with type 2 diabetes; thirty-two type 2 diabetes mellitus patients in the study, prescribed a diet or oral glucose-lowering agents, received GSE (600 mg/day) or placebo for four weeks in a double-blind randomized crossover trial, and the results indicate that GSE significantly improved markers of inflammation and glycaemia and improved a sole marker of oxidative stress in obese type 2 diabetic subjects at high risk of cardiovascular events over a four-week period, which suggests that it may have a therapeutic role in decreasing cardiovascular risk [36,37]. Berries are considered good sources of phenolic compounds, such as phenolic acids and flavonoids. Phenolic compounds from berry fruits as nutritional interventions could be considered a valuable tool to prevent the development of age-related neurodegenerative diseases by inflammation and decreasing oxidative stress [34].

According to Seeram (2008) [38], there is a wide range of observed biological properties associated with the phenolic compounds present in berry fruits, and they could be correlated to the type of individual phenolics rather than the total phenolic content. However, very little is known about the phenolic acid bioactive forms in vivo and the mechanisms by which they could contribute to the prevention of disease. Additionally, the little research on the biological effects of phenolic acids has ignored the issue of their possible concentrations in circulation after assimilation, such as the possibility of metabolism [39,40]. Hence, there is a need for more in-depth studies into the bioavailability and metabolism of these compounds, since the in vitro antioxidant activity does not reflect the in vivo biological activity. There is also a need to take into consideration the individual to individual variability for metabolism and the absorption of phenolic compounds. Further, it is known that many phenolic compounds are metabolized by the microbiota in the colon, leading to differences in the rate of metabolism and the products formed [41].

## 3. Type 2 Diabetes and Related Hypertension Complications

This section briefly describes the various aspects related to diabetes, including triggering factors, classification and related hypertension complications, focusing on the effective treatment offered at present for type 2 diabetes. The most important is to change the lifestyle (diet and exercise intervention). Finally, examples of phenolic compounds lowering blood pressure show the beneficial effects on type 2 diabetes and the related hypertension complications.

There are some elements, for instance smoking, sedentariness and unhealthy dietary habits consuming too much high calorie, low fiber and high saturated fat refined food that lead to the morbidity of type 2 diabetes and hypertension, complications, and so on. Certainly, the most important factors are sedentariness and unhealthy eating habits [42]. It has been reported that although healthcare has distinctly grown worldwide, with the development of the food industry, the abundance of food, especially high calorie, high saturated fat and low fiber food, such as puffed food, quick meals and drinks, has led to great over-consumption, in addition to sedentariness, bringing about the increase of diabetics, mostly in developed countries, as well as some developing countries, and patients with diabetes are ever more young and widespread. Diabetes is a growing public health concern all over the world. In 2010, at least 285 million adults were affected by diabetes, and that number is predicted to reach 439 million by 2030 [43,44].

The common forms of diabetes mellitus are type 1 and type 2 diabetes, the latter being the more prevalent form and generally appearing later in life. Insulin resistance and impaired insulin secretion lead to type 2 diabetes, which has the features of chronic metabolic disorder and high levels of blood glucose [45]. Regarding type 2 diabetes, because of the pancreatic β-cells’ imperfection, damage or insulin resistance, the total secretion of insulin is insufficient, and as a result, the blood glucose of diabetics is much higher than the ordinary level [46]. Scavenging free radicals is a way to reduce oxidative stress. The hydroxyl radical (OH) and Fe^2+^ are free radicals in the body. It has been shown that oxidative stress and inflammation can lead to the development of obesity-related insulin resistance. The free radical scavenging ability of gallic and protocatechuic acids is shown in Figure 4 and Figure 5. 

In addition, hyperglycemia and dyslipidemia can induce inflammatory responses, in the meantime producing free radicals, which lead to type 2 diabetes and cardiovascular complications [47]. Some research hinted that hyperglycemia had been reported to cause increased production of oxygen free radicals through glucose auto-oxidation and nonenzymatic glycation (Maillard reaction) processes. In vivo Maillard reaction courses are complex. The essence is that the protein on the pro nuclear group and the sugar and other substances generate advanced glycation end products (AGEs). AGEs can induce the proliferation of vascular smooth muscle cells, which is one of the mechanisms of coronary artery disease induced by diabetes [48]. As a result, they are likely to destroy the components of cells and tissue, such as proteins, DNA and lipids [49,50]. In pancreatic β-cells, hyperglycemia-induced oxidative stress plays a key role in developing diabetes [51,52]. Some of the polyphenolic compounds make the β-cells from hyperglycemia-induced and oxidative-induced damage. A well-known phenolic compound, resveratrol (3,4′,5-tri-hydroxystilbene), has the properties of improving impaired glucose tolerance, attenuating β-cell reduction and reducing oxidative stress in the islet, which is found in grapes, wine, grape juice, peanuts and berries [53]. There are experiments to show that in streptozocin (STZ)-induced diabetic rats, there were beneficial impacts on serum glucose and the viability of β-cells through attenuating oxidative stress, improving the natural antioxidant system and inhibiting lipid peroxidation after oral administration of phenolic-rich chestnut extract [54].

Hypoglycemic agents, like acarbose and miglitol, controlling α-glucosidase, can be found in most hospitals and are commonly prescribed for obesity and diabetes. The effective treatment offered at present for type 2 diabetes is via inhibiting the absorption of glucose through reducing starch hydrolysis by slightly inhibiting gland α-amylase and intensely controlling intestinal glucose absorption by beta-glucosidase enzymes. Though pharmacological treatment for metabolic syndrome can be offered, changing the lifestyle (diet and exercise intervention) ought to be considered [55]. Hence, some researchers have found that diet and exercise intervention can markedly reduce the morbidity of diabetes; particularly, uniting diet improvement with exercise, there was around a 10% reduction when the two were combined compared to diet alone. Modifying lifestyle factors, for example high-quality diets, are substantially related to a lower incidence of type 2 diabetes.

Excessive α-amylase inhibition can lead to undigested starch in the colon and consequently lead to stomach distention and discomfort. A novel process combining enzymatic debranching, melting and crystallization was developed to produce spherulites from short linear α-1,4-linked glucans (short-chain amylose (SCA)) with controlled enzyme digestibility. SCA was obtained by completely debranching waxy maize starch at 50 °C and 25% solids in 0.01 M sodium acetate buffer. The mixture was then heated to 180 °C followed by cooling and crystallization to form well-developed spherulites. Spherulites crystallized at low temperatures (4 and 25 °C) had a large size (5–10 μm), a B-type starch X-ray diffraction pattern, a lower melting temperature (70–110 °C) and a higher digestibility (Englyst method) compared to the spherulites crystallized at 50 °C, which had a small size (1–5 μm), an A-type diffraction pattern, a higher melting temperature (100–140 °C) and a lower digestibility. Intact spherulites along with small fragments were observed after digestion with a mixture of α-amylase and amyloglucosidase, indicating that digestion was not homogeneous and preferentially occurred in weak spherulites [56]. Some polyphenols have properties of regulating the pivotal pathways of carbohydrate metabolism and hepatic glucose homeostasis, including glycolysis, glycogenesis and gluconeogenesis, usually impaired in diabetes. Ferulic acid effectively inhibits blood glucose by promoting glucokinase activity, producing glycogen in the liver and increasing blood insulin levels. The study suggests that insulin-dependent diabetes mellitus is an autoimmune disease caused by selective destruction of pancreatic beta cells. Additionally, polyphenolic compounds can increases the catalytic activity of glucose phosphorylation; what is more, natural plants extracts, such as eugenol, can effect glucose metabolism. Phenolic compounds inhibit the expression of the inducible nitric oxide synthase gene and inhibit the activity of transcription factor NF-kB. Therefore, nitric oxide (NO) products can be prevented, and the protective effect of insulin-producing rat pancreatic cell line (RINm5F) and pancreas on insulin secretion and secretion and islet beta cell activity can be maintained. In addition, natural plants, like cinnamon extract, can activate glycogen synthase and insulin receptor kinase, increase in vivo glucose uptake, inhibit glycogen synthase kinase-3 beta, inhibit the dephosphorylation of the insulin receptor and improve insulin sensitivity.

Some recent research have indicated that phenolic compounds are capable of inducing the inhibition of α-amylase and α-glucosidase, hence, beneficial for the treatment of type 2 diabetes [57]. Some experiments have revealed that polyphenolic compounds are also capable of regulating postprandial blood glucose and inhibiting the development of glucose intolerance by promoting insulin response and attenuating the secretion of glucose-dependent insulinotropic polypeptide (GIP) and glucagon-like polypeptide-1 (GLP-1) [58,59]. Thus, natural α-amylase and α-glucosidase inhibitors from fruits and vegetables could offer a good strategy to control post-prandial hyperglycemia and provide benefits without the side effects present in most available drugs, such as abdominal distention, flatulence and possibly diarrhea [31,60]. Phenolic phytochemicals have also been found to have potential in the management of oxidative stress-linked chronic diseases, like diabetes, cancer and cardiovascular disease [20].

Dietary polyphenols also impact peripheral glucose absorbed in both insulin-sensitive and non-insulin-sensitive tissues. One research work indicated that phenolic acids excite the uptake of glucose with a similar method of absorption of metformin, as well as thiazolidinedione, the main common orally-taken hypoglycemic agent [61]. Some polyphenols are also capable of inducing phosphatidylinositide 3-kinase (PI3K) as a pivotal signaling approach for increasing the assimilation of glucose uptake [62]. Ford et al. (2001) reported a significant reduction in the risk of type 2 diabetes among female participants consuming five or more servings of fruits and vegetables per day compared with those consuming none [63]. Fogli-Cawley et al. (2007) studied [64] the association between the 2005 Dietary Guidelines for Americans (DGA) and the risk of metabolic syndrome. Those authors reported that the consumption of a diet following the DGA recommendations led to lower levels of many risk factors for metabolic syndrome along with reduced prevalence.

The beneficial effects of the consumption of coffee by type 2 diabetic individuals have been reported [65,66], especially due to the presence of chlorogenic acid. The possible explanation is that chlorogenic acid might interact with the absorption of glucose from the intestine by inhibiting of Na^+^-dependent glucose transporters, SGLT1 and SGLT2 [58,67]. Additionally, a similar principle of action to the pharmaceutical drug (acarbose) used in the treatment of type 2 diabetes was shown [68]. Consumption of high-polyphenol dark chocolate had the benefits of improving endothelial function in individuals with stage 1 hypertension, and the attenuating of serious transient hyperglycemia-induced endothelial dysfunction and oxidative stress in type 2 diabetic patients had been proven in human trials.

It has been reported that individuals affected by type 2 diabetes are associated with many risk factors, including hypertension, dyslipidemia and obesity [69]. The renin-angiotensin system is important for regulating blood pressure, water and electrolyte balance. Angiotensin I-converting enzyme (ACE) is an important enzyme involved in maintaining vascular tension by two different reactions that it catalyzes: conversion of the inactive angiotensin I into the powerful vasoconstrictor and salt-retaining angiotensin II and inactivation of the vasodilator bradykinin, which is conducive to lowering blood pressure. Inhibitors of the renin-angiotensin system (RAS) may enhance the effect of initial or early therapy of hypertension [70], which is a microscopic vascular complication of type 2 diabetes.

Suzuki et al. (2002) reported [71] an ACE inhibitory activity of chlorogenic acid with an improvement on the vasodilation in high blood pressure of rats. The effect of ferulic acid on blood pressure (BP) was investigated in spontaneously hypertensive rats (SHR), and these data suggest that the hypotensive effect of ferulic acid in SHR is associated with NO-mediated vasodilation. Suzuki et al. (2008) reported [72] the potential positive effect on hypertensive rats after administration of coffee. Furthermore, water-soluble extracts of green coffee bean, whose main component was chlorogenic acid, lowered blood pressure in mildly hypertensive patients [73]. Edwards et al. (2007) reported [74] that quercetin supplementation reduced blood pressure in hypertensive subjects. These authors suggested that there is a potential for the use of this phenolic compound as adjunct therapy in diet/lifestyle interventions in the control of blood pressure in hypertensive individuals. Furthermore, Nakamura et al. (2002) reported [75] a positive effect in the vasorelaxation of rat aorta after administration of a black currant concentrate. The consumption of high-flavonoid sweet juices (hybrid between grapefruit and pomelo) had significant beneficial effects on the reduction of blood pressure than low-flavonoid sweet juices [76]. However, Nettleton et al. (2007) reported [77] that the ingestion of flavonoids and flavonoid-rich foods was not associated with a reduction in the risk of type 2 diabetes in postmenopausal women. Only the regular consumption of red wine among these women was more related to a decrease in the incidence of type 2 diabetes when compared to women that did not consume red wine.

The risk of developing type 2 diabetes could be reduced by the changing of dietary patterns and particularly higher consumption of fruits and vegetables. The consumption of vegetables, legumes and fruits was significantly associated with reduced risks of all-cause mortality, but exclusive of cancer, in a European diabetic population [35]. Furthermore, these authors suggested that diabetic patients could benefit from a diet rich in fruits and vegetables. During an intervention in adults with type 2 diabetes, the participants selected more servings of whole fruits and nonfat dairy products, fewer servings of tomato-based products and vegetable fats, indicating that education and availability of information could help consumers make greater dietary changes.

## 4. Health Benefits of Berry Phenolics for Potential Type 2 Diabetes Management

Many foods and herbal extracts have been reported as having active effects on the diabetic patient. People began to use herbs and their active ingredients to prevent the treatment of diabetes and its complications. The change of the diet structure is gradually being applied to the diet [78]. For example, a diet abundant in fruits and vegetables can reduce the risk of type 2 diabetes, illustrating the beneficial effects on type 2 diabetes with different berries, such as strawberries, raspberries, blueberries and black currants. These are the main contents of the part below.

As discussed previously, α-glucosidase and α-amylase are well-known enzymes in the management of hyperglycemia linked to type 2 diabetes [79]. Many foods and herbal extracts have been reported as having a positive effect in diabetic glycemic control using traditional medicine [80]. Polyphenol withdrawn from red wine, ethanol or a combination of both can control the health of streptozotocin-induced diabetic rats [81]. What is more, because of the antioxidant properties, cereal, fruit and vegetable fiber intake is associated with lower risk of ischemic heart disease and death, especially among old people.

In addition, many studies have reported the beneficial effects on hypertension by polyphenol-rich extracts by retarding the development of hypertension and by normalizing blood pressure [82]. In this condition, some foods and herbs could have the potential to treat hypertension, especially for patients with borderline to mild high blood pressure.

Berries are considered good sources of phenolic compounds, such as flavonoids and phenolic acids. Beyond that, berry fruits are commonly consumed fresh and as derived products, such as canned fruits, yogurts, juices and jams. Many studies have reported the benefits of berry consumption against several types of human cancers [38,83] and metabolic syndrome [84].

The polyphenol components of berries inhibit α-glucosidase and α-amylase enzymes, resulting in reduced blood glucose levels after starch-rich meals [85]. McDougall et al., (2008) studied the potential inhibitory activity of strawberries, raspberries, blueberries and black currants on α-glucosidase and α-amylase enzymes [86]. These authors reported that blueberries and black currants had the highest α-glucosidase inhibitory activity, and strawberries and raspberries had the highest α-amylase inhibitory activity. According to Song et al., (2005) [87], reducing risk for type 2 diabetes was associated with high consumption of berries and apples and a diet rich in fruits and vegetables. 

High anthocyanin-containing fruit extracts are obtained from currant, blueberry, raspberry and strawberry, and good α-glucosidase inhibition by these extracts was observed [85,86]. However, Cheplick et al., (2007) reported [88] high α-glucosidase inhibitory activity for a yellow raspberry cultivar among black, red and yellow raspberries, suggesting that the α-glucosidase may be influenced more by specific anthocyanins rather than the actual amount of the overall total plant phenolics. α-amylase inhibitory activity in different raspberry cultivars might be due to some specific phenolics [88], since many fruits, including grapes, raspberries and strawberries, are known to contain high levels of soluble tannins, and these fruits have α-amylase inhibitory properties [85,86]. 

Wilson et al., (2008) suggested [89] that cranberry juice could represent an attractive means for increasing fruit intake and simultaneously affording positive health benefits. Apostolidis et al., (2007) reported [90] that cranberry-enriched cheese had the highest α-amylase and α-glucosidase inhibitory activities among herb, fruit and fungal-enriched cheeses by in vitro studies. Chambers and Camire (2003) [91] evaluated the ingestion of capsules filled with cranberry juice concentrate by adults with type 2 diabetes for 12 weeks. No significant difference in the blood glucose levels was observed. However, these authors suggested that more concentrated products might have benefits since the commercially-available cranberry juice cocktails contain only 27%–31% cranberry juice.

The potential effects on the in vitro inhibition of α-amylase and α-glucosidase enzymes from different Brazilian strawberry cultivars were shown [92]. These authors reported that strawberries had high α-glucosidase and low α-amylase inhibitory activities, suggesting these fruits as good sources for the potential management of hyperglycemia linked to type 2 diabetes as a part of an overall diet. Apostolidis et al., (2006) reported [93] that the combination of cranberry with oregano, which had higher rosmarinic acid content, contributed to the high antioxidant activity and total phenolic content in the extracts, suggesting potential relevance for type 2 diabetes and related hypertension.

The dietary management of hyperglycemia linked to type 2 diabetes through foods that have high α-glucosidase and moderate α-amylase inhibition has been suggested. This is due to the fact that excessive α-amylase inhibition can lead to undigested starch in the colon and consequent stomach distention and discomfort [56]. Hence, berry fruits with low α-amylase and high α-glucosidase inhibitory activities could be considered as good potential candidates as a part of an overall dietary design to manage early stages of hyperglycemia linked to type 2 diabetes.

## 5. Health Benefits of Other Phenolics for Potential Type 2 Diabetes Management

The following content is about the beneficial effects of some other phenolics that are exacted from pears, pomegranate peel, *Phaseolus vulgaris* and *Cynara scolymus* (artichoke) for potential type 2 diabetes management.

Pear (*Pyrus* spp.) is one of the most popular fruits, consumed as both fresh fruit and fruit products worldwide. Generally speaking, pear peel contains more nutrient components than its pulp. Pear has been reported as a potential source for polyphenols and triterpenes [94]. Meanwhile, polyphenol plant extracts possess potential key enzyme of type 2 diabetes (α-glucosidase and α-amylase), as well as hypertension disease (angiotensin-converting enzyme) [30,95].

Oboh et al., (2012) sought to investigate the inhibitory effect of phenolic extract from avocado (*Persea americana*) leaves and fruits on some key enzymes linked to type 2 diabetes (α-amylase and α-glucosidase) [95]; and sodium nitroprusside (SNP)-induced lipid peroxidation in rats’ pancreas in vitro. The result showed that the leaves and fruit of avocado inhibit both α-amylase and α-glucosidase activities in a dose-dependent manner. However, the peel had the highest α-amylase inhibitory activity, while the leaf had the highest α-glucosidase inhibitory activity as revealed by their IC_50_ value [95].

*Punica granatum* (Pomegranate) is rich in flavonoids, such as flavonols, flavanols and anthocyanins, hydrolysable tannins, such as ellagitannins and gallotannins, condensing tannins, such as proanthocyanidins, and organic and phenolic acids [30]. The efficiency of pomegranate in type 2 DM could be explained by decreasing the lipid peroxidation and oxidative stress through different mechanisms, such as enhancing the antioxidant activity of some enzymes, reducing reactive oxygen species (ROS), inducing metal chelating activity and inhibiting or activating transcriptional factors, such as PPAR-γ and nuclear factor kappa B [96]. Recently, pomegranate wine (2.0 μg/mL) was found to inhibit NF-kappa B activation in cultured vascular-endothelial cells, and studies performed on human acute monocytic leukemia cell line-1 to differentiate into macrophages showed that the traditional anti-diabetic effect of the methanolic extract from pomegranate flowers (PFE) at 500 mg kg^−1^·day^−1^ is due to the enhancement of peroxisome proliferator-activated receptor (PPAR)-γ, a transcription factor that plays an important role in carbohydrate metabolism.

According to an experimental study in diabetic rats with non-alcoholic fatty liver disease, the protective effects of polyphenols from pomegranate flowers could be explained by the increase of liver PON1 mRNA and protein expression that enhanced the body antioxidant capacity and reduced immunoreactive insulin to ameliorate the rat hepatic steatosis.

The common bean (*Phaseolus vulgaris* L.) with a high content of flavonoids, such as flavonols, flavanones, bioflavonoids, flavones, isoflavones, isoflavans, anthocyanins, pterocarpans and coumestans, is one of the most well-known hypoglycemic herbal drugs. Experimental evidence in diabetic animals indicates that *Phaseolus vulgaris* extracts are effective in reducing lipid accumulation, glycemia, appetite, weight and carbohydrate absorption. *Phaseolus vulgaris* made serum levels of HDL-cholesterol, plasma insulin, glutathione, vitamin C and the antiatherogenic index in diabetic rats normal. Another study in streptozotocin-induced diabetic rats concluded that cooked common beans (*Phaseolus vulgaris* L.) can protect against pancreatic beta-cell damage. Various clinical studies proved that *Phaseolus vulgaris* contains an alpha-amylase inhibitor, which will inhibit or delay digestion or effect carbohydrate absorption [97,98,99].

Anthocyanins are considered as modulators of adipose tissue metabolism. This study shows that anthocyanins have a significant potency for anti-obesity and to ameliorate fat cell dysfunction, as well as the secretion of adipocytokines in insulin resistance, increasing β-oxidation and reducing fat accumulation in adipose cells [100]. *Corchorus olitorius* leaf extracts are a type of polyphenol (free and bound) with inhibitory activities influencing key enzymes (α-amylase and α-glucosidase) linked to type 2 diabetes and through the inhibitory activities against type 2 diabetes. Inhibition of α-amylase and α-glucosidase activities by these extracts coupled with high antioxidant activity could be part of the mechanism by which *C. olitorius* leaf exhibits its anti-diabetic properties by preventing hyperglycemia and oxidative stress and damage to biomolecules (lipids, proteins, DNA). In addition, ACE inhibition may explain the antihypertensive properties. These characteristics can be attributed to its phenolic constituents, such as caffeic acid, chlorogenic acid and isorhamnetin, which are abundant in the leaf. However, this is an in vitro finding with possible physiological implications [95].

*Cynara scolymus* (artichoke) is a perennial herb of the Compositae family, being wide-ranging and considered for its anti-hypercholesterolemic and glucose-lowering effects. An artichoke globe presents no fat, 170 mg of potassium and is rich in vitamin C, cynarin, orthophenole derivates, magnesium, folate and dietary fiber [101]. Recent studies indicated that artichoke flavonoids upregulated nitric-oxide synthase expression in endothelial cells [102]. NO synthesis can be adjusted through the activity of endothelial nitric-oxide synthase (eNOS); the eNOS can generate both nitric oxide (NO), causing blood vessels to dilate, and superoxide, making blood vessels shrink. Therefore, eNOS, having abilities, such as antithrombotic, anti-atherosclerotic and the antihypertensive properties of endothelial NO, is important in regulating vascular function. A study on the endothelial hybrid cell line (EA.hy 926) cells, a cell line derived from human umbilical vein endothelial cells (HUVECs), has found that artichoke leaf extract (ALE), containing large amounts of polyphenolic compounds, can increase eNOS promoter activity in a concentration-dependent manner and, so, influence NO synthesis. That is the way that artichoke flavonoids upregulated nitric-oxide synthase expression to regulate vascular function. Another experimental study in normal and obese rats showed that artichoke extract lowers blood sugar [103]. A clinical study proved that *Cynara scolymus* supplements are also effective on glucose metabolism in patients with impaired fasting glycemia.

Plant polyphenols can improve the endogenous antioxidative system, effectively prevent oxidative damage and improve oxidant-antioxidant balance. The research on green tea shows that it contains six primary catechins as the polyphenolic compounds that are the most common in this field; and these bioactive components reduced lipid peroxidation and increased plasma total antioxidant capacity. Stress-sensitive signaling pathways, preoxidant enzymes and the induction of antioxidant enzymes, including superoxide dismutase, catalase and glutathione peroxidase, were also decreased [104]. In conclusion, one of the pathogenic mechanisms that describes the increase and progression of micro- and macro-vascular complications in diabetes is oxidative stress; increased production of free radicals and an impaired antioxidant defense system in diabetes induce the state of oxidation/antioxidant imbalance [105]. Inhibition of these oxidative processes could prevent the onset and development of long-term diabetic complications [106].

## 6. Some Suggestions on Natural Phenolic Compounds and Perspectives

Nowadays, more and more people are beginning to pay attention to gestational diabetes mellitus (GDM). A balanced diet can help prevent and manage several human diseases and metabolic disorders. Examples verify that a higher intake of phenolic compounds and flavonoids from food (vegetables and fruits) is consistently associated with a significantly lower risk of the GDM [107]. In addition, these studies have found positive results for resveratrol on treating diabetes, cardiovascular disease or heart disease [108]. Several lines of evidence also show that high antioxidant content fruits and vegetables may reduce the risk of certain chronic diseases, such as type 2 diabetes, and are associated with the increased risk of microvascular and macrovascular complications. Therefore, phenolic compounds of fruits and vegetables have great potential for the management of type 2 diabetes by controlling hyperglycemia, its macrovascular complications, such as hypertension, and microvascular complications linked to cellular oxidative breakdown. There is usually a large number of different substances and a distribution of conjugated forms of phenolic compounds along with functionality among berry fruits that could be taken advantage of for planning compatible functional advantages for the management of the metabolic syndrome of the chronic disease state. Especially, berry fruits could be determined as a part of a general healthy diet for the management of postprandial hyperglycemia, due to their ability to inhibit α-glucosidase simultaneously with low inhibition of α-amylase, which probably lead to less adverse effects. Further, the similar profiles of bioactive phenolic compounds also have potential, which avoid cellular oxidative breakdown linked to macro- and micro-vascular complications, as well as hypertension, respectively. Natural plant phenolic compounds present in a wide variety of natural plants have played an effective role in the treatment of type 2 diabetes, so it will be a major research direction that should be explored in today’s society.

Type 2 diabetes is a polygenic metabolic disease characterized by a concomitant impairment of the metabolic functions of several organs. The anti-diabetic effects of phenolic phytochemicals are similar due to the integration of several complementary mechanisms. It is a difficult task to elucidate the mode of actions of these polyphenolic compounds given their large numbers and the specific regulation of their bioavailability as determined by their absorption and biotransformation. Due to the in vitro inhibitory effects of plant-based food extracts (grape seed, green tea and white tea) and their constituent flavan-3-ol monomers (catechins) on α-amylase and α-glucosidase activity, two key glucosidases required for starch digestion in humans [109], the potential role of natural phenolic compounds is discussed.

Type 2 diabetes is a cluster of metabolic disorders. And type 2 diabetes is associated with other pathogenic conditions, including subclinical inflammation and oxidative stress. The pathogenic conditions result in long-term diabetes complications and insulin resistance. The escalating tendency in the prevalence rate of diabetes complications hints that recent medical treatments for the management of diabetes are not adequate, and the use of additional treatments, which consist of their nutraceuticals and functional foods, could raise the validity of diabetes management. Polyphenols in plants, which include phenolic acids, lignans, stilbenes and flavonoids, based on in vitro research, clinical trials and some animal models, have been proposed as efficient supplements for diabetes management and as preventive of its long-term complications. Further investigations making use of human clinical research are required to affirm the useful results of polyphenolic compounds as additional treatments for diabetic patients.

## 7. Conclusions

Summing up from the context, high levels of antioxidant compounds and a diet abundant in fruits and vegetables could potentially reduce the risk of type 2 diabetes and the associated increased risk of microvascular and macrovascular complications. Therefore, within the context phenolic antioxidants, those available from berries have excellent potential for managing type 2 diabetes through the control of hyperglycemia and its macrovascular complications, such as hypertension and microvascular complications linked to cellular oxidative breakdown. High phenolic antioxidant activity suggests that certain phenolic compounds are present in select species, which could prove to be beneficial towards human health if included as part of food designs for a healthy diet. This review provides a biochemical rationale for clinical studies on the functional benefits of fruits and vegetables, which could further be applied in in vivo studies for the development and innovation of therapeutic strategies, to prevent and manage type 2 diabetes. 

## Figures and Tables

**Table 1:** Fasted body weight, body weight gain, tissue weight, ratio of tissue-to-body weight and hemoglobin A1 of rats after the experiment for four weeks ^a^ [23].

Wt, Wt Gain and Ratio	Normal	Control	Crude Extract of *V. dilatatum* Fruit-Low	Crude Extract of *V. dilatatum* Fruit-High
Final body wt (g)	337.8 ± 17.7	238.2 ± 22.6 ^c^	239.5 ± 19.4 ^c^	244.7 ± 12.6 ^c^
Body wt gain (g)	142.4 ± 14.5	55.2 ± 16.2 ^c^	56.4 ± 14.2 ^c^	60.1 ± 7.7 ^c^
Liver wt (g)	10.65 ± 0.73	8.96 ± 0.72 ^c^	8.30 ± 0.64 ^c^	8.02 ± 0.83 ^c^
Kidney wt (g)	2.462 ± 0.189	2.208 ± 0.263	1.895 ± 0.310 ^c^	1.846 ± 0.359 ^c^
Pancreas wt (g)	0.946 ± 0.180	0.597 ± 0.076 ^c^	0.632 ± 0.151 ^c^	0.664 ± 0.081 ^c^
Tissue-to-body wt ratio ^b^ Liver (g/100 g)	3.162 ± 0.294	3.771 ± 0.216	3.485 ± 0.378	3.288 ± 0.434
Kidney (g/100 g)	0.730 ± 0.071	0.930 ± 0.111 ^c^	0.789 ± 0.093	0.751 ± 0.115
Pancreas (g/100 g)	0.279 ± 0.042	0.252 ± 0.032	0.266 ± 0.067	0.271 ± 0.032
Hemoglobin A1 (%)	2.16 ± 0.18	4.27 0.60 ^c^	3.29 ± 0.84 ^c^	2.65 ± 0.36 ^d^

^a^ Rats were fasted for 20 h after the experimental period of 4 weeks. Data represent the mean ± SD of five rats. ^b^ Tissue weight per 100 g of body weight. Significant difference is indicated from the normal ^c^ and control ^d^ groups at *p* < 0.05.

**Table 2:** Enzyme inhibitors from plant origins and their corresponding inhibition of enzymes in starch digestion.

Plant Origin	Enzyme Inhibitor	Enzyme Targets	References
Pakhanbhed *(B. ciliata)*	(−)-3-*O*-galloylepicatechin, (−)-3-*O*-galloylcatechin	Pancreatic α-amylase, maltase	Bhandari et al., 2008 [26]
Persimmon *(Diospyros kaki)*	Proanthocyanidin ((−)-epigallocatechin, (−)-epigallocatechin-3-*O*-gallate, (−)-epicatechin, (+)-catechin, (−)-epicatechin-3-*O*-gallate)	Pancreatic α-amylase	Kawakami et al., 2010 [28]
Finger millet *(E. coracana)*	Naringenin, kaempferol, luteolin glycoside, apigenin, daidzein, caffeic acid, ferulic acid, syringic acid	Pancreatic α-amylase, α-glucosidase	Shobana et al., 2009 [29]
Raspberries *(Rubus idaeus)*	Ellagic acid, catechin, pelargonidin-3-rutinoside, cyanidin-diglucoside	α-Glucosidase	Zhang et al., 2011 [30]
Black myrobalan *(T. chebula)*	Chebulanin, chebulagic acid, chebulinic acid	Maltase	Gao et al., 2007 [31]
Black/bitter cumin (*C. anthelminticum*)	Caffeic acid, ellagic acid, ferulic acid, quercetin, kaempferol	Maltase, sucrase, salivary α-amylase	Ani and Akhilender Naidu., [32]
